# Potential use of cold plasma treatment for disinfection and quality preservation of grape inoculated with *Botrytis cinerea*

**DOI:** 10.1002/fsn3.3876

**Published:** 2023-12-07

**Authors:** Ali Khalaj, Ebrahim Ahmadi, Sohiela Mirzaei, Fahiemeh Ghaemizadeh

**Affiliations:** ^1^ Department of Biosystems Engineering, Faculty of Agriculture Bu‐Ali Sina University Hamadan Iran; ^2^ Department of Plant Protection, Faculty of Agriculture Bu‐Ali Sina University Hamadan Iran; ^3^ Department of Horticultural Science, Faculty of Agriculture Bu‐Ali Sina University Hamadan Iran

**Keywords:** *Botrytis cinerea*, cold plasma, postharvest quality, *Vitis vinifera*

## Abstract

Gray mold caused by *Botrytis cinerea* is a serious disease of grape (*Vitis vinifera*) during storage. The aim of this study is to evaluate the effect of atmosphere cold plasma (a novel and nonthermal technology) on inactivation of *B. cinerea* and preservation of chemical, physical, and mechanical characteristics of grape inoculated with *B. cinerea*. Herein, different time of cold plasma (0, 10, 20, and 40 s) was firstly considered to be the main factors, besides different storage time (1, 2, 3, 4, and 5 weeks) at 4°C. According to the results, plasma treatment exhibited inhibitory effect on gray mold percentage and microbial load of *B. cinerea* (log CFU g^−1^) during postharvest storage. So, in the last week, the gray mold percentage and microbial load in the control were 100% and 3.6 log CFU g^−1^, and in 40‐s plasma were 4.5% and 2.53 log CFU g^−1^, respectively. Although the minimum infection and microbial load were observed in 40‐s plasma, better postharvest quality preservation was observed in short‐time cold plasma treatment (≤20 s). Forty‐second plasma caused fruit tissue destruction and negatively decreased mechanical indices (Emod: 0.0028, Fmax = 1.78, and *W* = 3.18) and weight loss (91.9) in comparison with ≤20‐s plasma, in which mechanical indices (Emod =0.0077, Fmax = 3.6, and *W* = 10.06) and weight loss (1/1) were higher. The long‐time cold plasma treatment (40 s) had also maximum effects on color changes (10) and surface temperature (2.8°C). Although the highest TSS and TA were observed in 40‐s Plasma, but different time of plasma treatments had no effect on pH. Altogether, these results indicate that the short‐time cold plasma treatment can inactivate *B. cinerea* on grape berries and preserve crop quality properties.

## Graphical abstract

A plasma application probe was designed and fabricated specifically for grape berries. Plasma treatment effectively reduced the infection percentage and its microbial load. Crop quality preservation and better infection control were observed in plasma treatment.

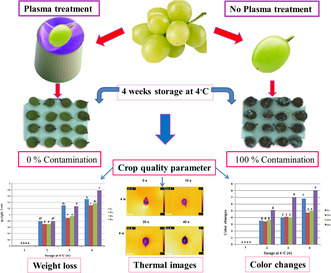

## 1 INTRODUCTION

Grapes are a major horticultural crop with a high nutritional value in the world. The appropriate geographic and climatic conditions in Iran make it one of the most important regions for grape cultivation and a major producer of this crop in the world (FAO, 2020).

Grapes produce more postharvest losses due to their high water content and specific physiological nature. Fungal rots are among the most important factors of postharvest losses in grapes. These diseases are prevalent in most parts of the world and cause high losses (Kulakiotu et al., 2004). Grapes are very sensitive to fungal penetration because they contain low acidity, high water, and nutritional compounds, which results in crop rot and spoilage during ripening and storage, making them unusable (Meng et al., 2010; Moss, 2002).

Fungi of *Botrytis* genera fall into the major fungi that cause the bunch rot of grapes, the signs of which may present as bunch stalk rot, berry cracking, and gray mold development (caused by *Botrytis cinerea*) on berries. These complications gradually cause water loss, crushing and fall of berries, and finally loss of transport and storage properties (Keller et al., 2003). According to Dean et al. (2012), *Botrytis* has the second rank among 10 plant pathogenic fungi in terms of economic loss.

Different methods include fumigation of bunches with sulfur dioxide and storage with a modified atmosphere (Artes‐Hernandez & Aguayo, 2004; Droby & Lichter, 2007; Duarte‐Sierra et al., 2016; Lu et al., 2013). Low temperatures in springhouses also prevent the growth of microorganisms and enzymatic activities and increase crop shelf life (Erkmen & Bozoglu, 2016). The use of coating and film technology can also effectively prevent the growth of yeasts and molds and preserve crop quality (Mannozzi et al., 2018).

However, each of the mentioned methods suffers from disadvantages. Grape fumigation with sulfur gas is harmful to human health (De Simone et al., 2020). The effectiveness of using atmospheric control storage, modified storage, and atmospheric modification packaging on fungal decay control and maintaining grape quality depends on the type of variety, storage temperature, and especially gas concentration (Himelrick, 2003; Sanchez‐Ballesta et al., 2020). CO_2_ is effective in controlling fungal decay, but a high concentration of CO_2_ (>10%) reduces the taste quality and increases cluster browning (Crisosto & Mitchell, 2002; De Simone et al., 2020). Springhouse construction with chilling equipment is costly and it is difficult to create rapid, homogenous, and constant chilling. The problems of coating and film techniques include instability, easy degradation, and high hydrophobicity of compounds (Olivas & Barbosa‐Cánovas, 2009).

Since traditional methods lack the necessary efficiency, it will be very effective to propose modern techniques that fight against microorganisms and feature such advantages as maintaining crop quality, increasing crop shelf life, maintaining health, and high economic benefit. A technique of this type includes using nonthermal (cold) plasma (NTP) technology to eliminate food microorganisms (Bourke et al., 2018; Ekezie et al., 2017; Mir et al., 2020; Moreau et al., 2008).

NTP is a new and rapid technique which can work continuously at atmospheric pressure (Dasan & Boyaci, 2018; Hong et al., 2009; Martens, 2010; Massines et al., 1998; Segura‐Ponce et al., 2018). The efficiency of NTP technique in the disinfection of a wide range of microorganisms (Bacteria, yeast, and fungi) and in many fruits, vegetables, fruit juice, and nuts has been reported (Wiktor et al., 2020). Also, research has shown that cold plasma inactivates *Botrytis* in some agricultural products (Dong & Yang, 2019; Rana et al., 2020).

The effect of cold plasma on the quality characteristics of the product during the postharvest period has also been investigated. In blueberries, cold plasma for less than 15 min and after 10 days caused fruit darkening and lipid peroxidation reduction but had no effect on the total anthocyanin content, pH, and firmness (Hu et al., 2021). In strawberries, cold plasma (15 min) with packaging after 5 days of storage at 25°C did not affect the pH, TSS, and moisture content of the fruit (Rana et al., 2020).

To our knowledge, no previous researches have been found regarding the effect of cold plasma on grape. This investigation aims to scrutinize plasma treatment efficiency in the reduction of infection with *Botrytis* fungi, along with examining the physical, chemical, and mechanical properties of grape cv. Fakhri. Since fungal diseases are among the major spoilage and economic loss in this grape, results obtained from this study will demonstrate the feasibility of NTP as an effective method in grape shelf life and storage.

## 2 MATERIALS AND METHODS

### 2.1 Preparation of plant material

In this study, samples of grape cv. Fakhri were harvested from orchards in Hamadan province in the early morning and kept in cold storage at 1°C for 24 h. Healthy and uniform bunches with no signs of mechanical damage and fungal rot were chosen for the treatments.

### 2.2 Plasma treatment

#### 2.2.1 Plasma generator design and manufacture

**FIGURE 1:**
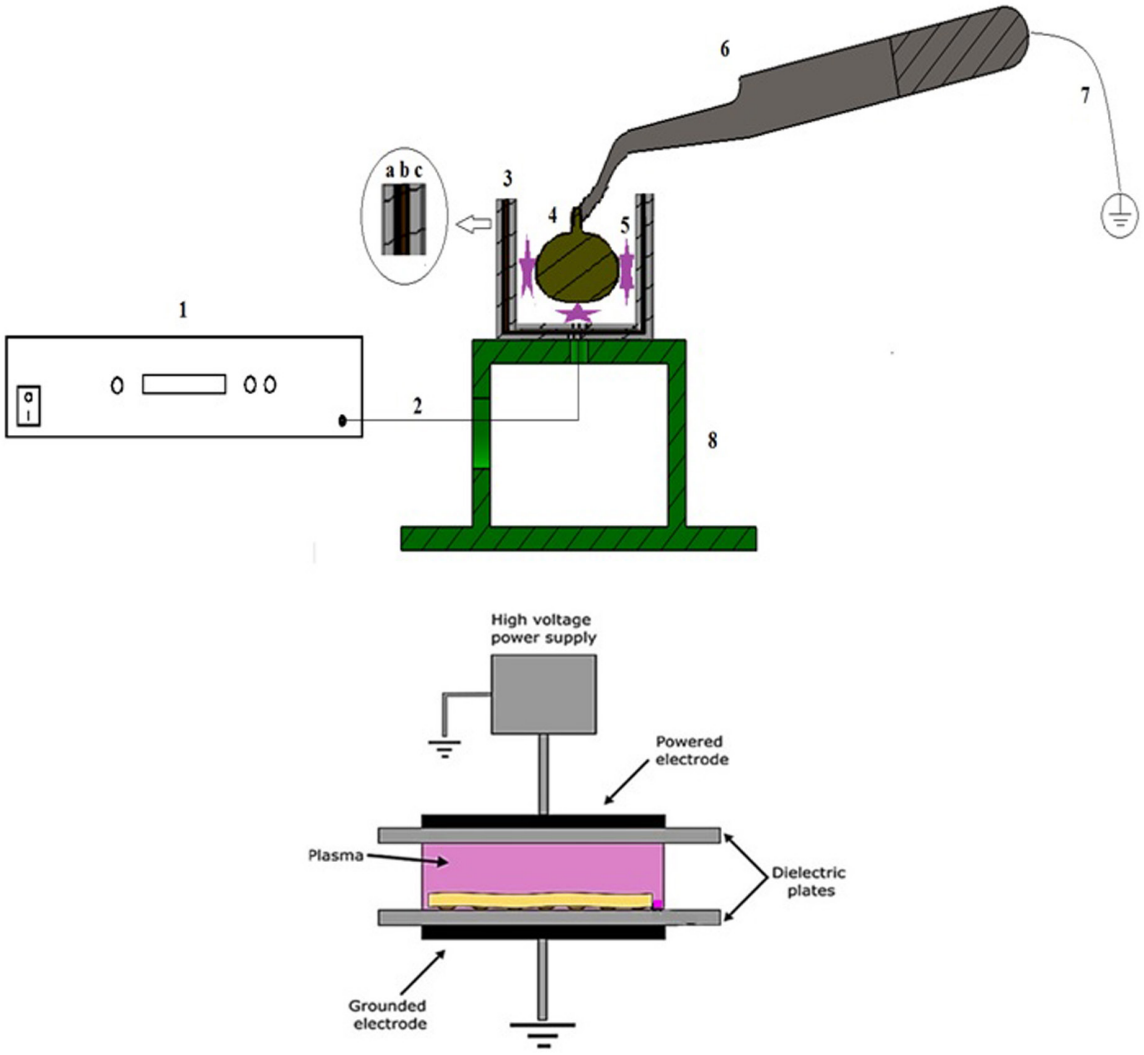
Schematic diagram of cold plasma device and probe designed at the institute of Biosystem lab (Bu‐Ali Sina University). 1: power supply, 2: wire transfer plasma, 3: probe consisted of dielectric (a and c) and copper electrode (b), 4: grape sample, 5: plasma showed by star, 6: second electrode for plasma discharge, 7: earth wire, 8: stand.

A plasma generator was first designed and manufactured in this study. A plasma application probe was also designed and fabricated specifically for grape berries (Figure 1a,b). The probe electrode structure consisted of a 20‐mm glass cylinder (dielectric) the exterior wall of which was wrapped with a copper sheet. A metal forceps was considered as the second electrode connected to the earth with a wire. This forceps was used to keep the grape berry in the glass cylinder and plasma application. An alternating voltage of 10 kV and 8.4 kHz frequency was applied to the copper electrode to produce uniform plasma inside the glass cylinder. A high voltage needed for plasma generation was provided using a frequency‐maker circuit consisting of a clock, MOSFET, and a transformer. The induction of a high voltage in transformer's secondary solenoid requires a high current to pass through its primary solenoid. Thus, a primary wave with an adjustable frequency is first created by the clock and then inserted into the MOSFET. Next, it enters transformer's primary solenoid with a high current and, finally, a high voltage is generated in the secondary solenoid.

#### 2.2.2 Preparation and artificial inoculation of samples with a fungal spore suspension

Healthy bunches were separated and washed with tap water for 1 min. Grape berries were then separated cautiously together with part of the fruit peduncle without damage to the tissue. The separated grape berries were sterilized with 1% sodium hypochlorite under a laminar hood for 2 min. The berries were washed thrice with sterile distilled water to remove the disinfectant residue from their surfaces. After washing, sterilized samples were dried under a laminar hood using paper filters and divided into two parts. The first part was considered healthy samples and later used as the control treatment (without fungal inoculation). The second was immersed in a *Botrytis* spore suspension at 10^6^ spores/mL. Finally, all samples were dried separately on paper filters and the second part samples were prepared for plasma treatment.

#### 2.2.3 Plasma treatment of samples

The second part samples underwent plasma treatment at three levels of 10, 20, and 40 s. To this aim, the plasma generator, containers for sample storage, and the forceps were disinfected with 70% ethanol. To reduce the spread of secondary infections, plasma was applied under a disinfected hood. After the plasma treatment, a Plexiglas mesh screen was made and placed in transparent disposable containers to prevent the contact of berries with each other and infection spread. The berries were then placed in these mesh spaces and kept at 4°C. The traits were evaluated for both control and Botrytis‐inoculated berries once weekly for 35 days.

### 2.3 Grape quality assessment after plasma treatment

#### 2.3.1 Infection percentage

To evaluate fungal infection, the infection percentage and microbial load were calculated by the visual observation of infected berries in each treatment based on the following equation.

$$\text{Infection percentage}=\left(N2/N1\right)*100,$$

N1 = Total berries, N2 = Number of infected berries.

To calculate microbial load at each sampling stage, three grape berries were (10 ± 2 g) separated from each treatment and dropped in a falcon containing 10 mL of sterile distilled water. The falcon was vortexed slowly, and 100 μL of the solution was cultured in a potato‐dextrose‐agar medium. The cultured samples were incubated at 22–24°C, and colonies were calculated after 12 h. Microbial count (microbial load) was expressed as the logarithms of CFU per gram (log CFU g^−1^).

#### 2.3.2 Physical properties

Fruit weight loss was measured by weighing the fixed fruits at the start of the experiment and then at each stage using a digital balance (0.01 precision). The weight loss percentage was obtained by the following formula:

$$\text{Weight loss percentage}=\left(\text{W1}-W2\right)/W1^{*}100,$$

W1 = Berries weight before storage, W2 = Berries weight during storage.

The color of samples was measured by the L.a.b method using a digital colorimeter (hp‐200). L*, a*, and b* values obtained from the device were recorded for three randomly selected and coded samples from each treatment. The color difference value (Δ*Ε*) was determined with the following formula:

$$\Delta E=\sqrt{{\Delta L}^{2}+{\Delta a}^{2}+{\Delta b}^{2}}$$

Δ *L* = lightness, Δ *a* = red–green, Δ *b* = yellow–blue.

#### 2.3.3 Mechanical properties of samples

The crop mechanical properties were evaluated with the puncture test using a food tester (Bbt1‐Fro.5th.D14; Zowick/roell) by a planar probe of 5 mm at 300 mm/min. In this test, the probe penetrated to a known depth (15% of the sample diameter), and a power–displacement curve was recorded by the device. The modulus of elasticity, changes in penetration power, and penetration level were measured in each test.

#### 2.3.4 Thermal imaging

During storage, grape thermal imaging was examined using an infrared temperature evaluation system consisting of a thermal camera (FLIR T420, the USA) with the following specifications:Field of view: 19° × 20°, Temperature sensitivity: 0.045°C, Detector and spectral range: 5.7–13 μm, Frame rate: 60 Hz, Screen display: 3.5 inch.

To prevent environmental fluctuations, all samples were imaged at 4 p.m. in similar conditions of the laboratory and a fixed device setting (similar light level, fixed background, and fixed tester) at 25 ± 0.5°C.

#### 2.3.5 Chemical properties (soluble solids, pH, and titratable acidity)

Total soluble solids (TSS) were determined with an Atago refractometer (PAL‐2, Japan) based on degrees Brix at 25°C. The juice pH value was measured using a pH meter (PHS3‐W3B) with 0.01 precision. Before each measurement, the device was calibrated with buffers 4 and 7 (Tyl & Sadler, 2017). To measure the total fruit acid using the titration method, 2 mL of the juice was homogenized with 10 mL of distilled water and titrated with 0.1 N NaOH to reach a pH of 8.2 ± 0.1. The titratable acidity (TA) was calculated with the following formula, and its value was expressed in the predominant fruit acid percentage (citric acid) (Tyl & Sadler, 2017).

$$\mathrm{TA}=\left(t\times m\times 75\right)/v,$$

*m* = molarity of NaOH, *t* = titer of NaOH required (mL), *v* = volume of sample used (mL).

### 2.4 Statistical analysis

All experiments were performed for three replicates. The experimental data were expressed as the mean ± standard deviation (SD). Data were analyzed by one‐way analysis of variance (ANOVA) and mean values were compared with Duncan's multiple‐rang test. The ANOVA was conducted using SAS software (9.4 M7version) (Rodriguez, 2011). Significant differences were identified with a confidence level of *p* ≤ .05.

## 3 RESULTS AND DISCUSSION

### 3.1 Evaluation of spoilage and microbial load

**FIGURE 2:**
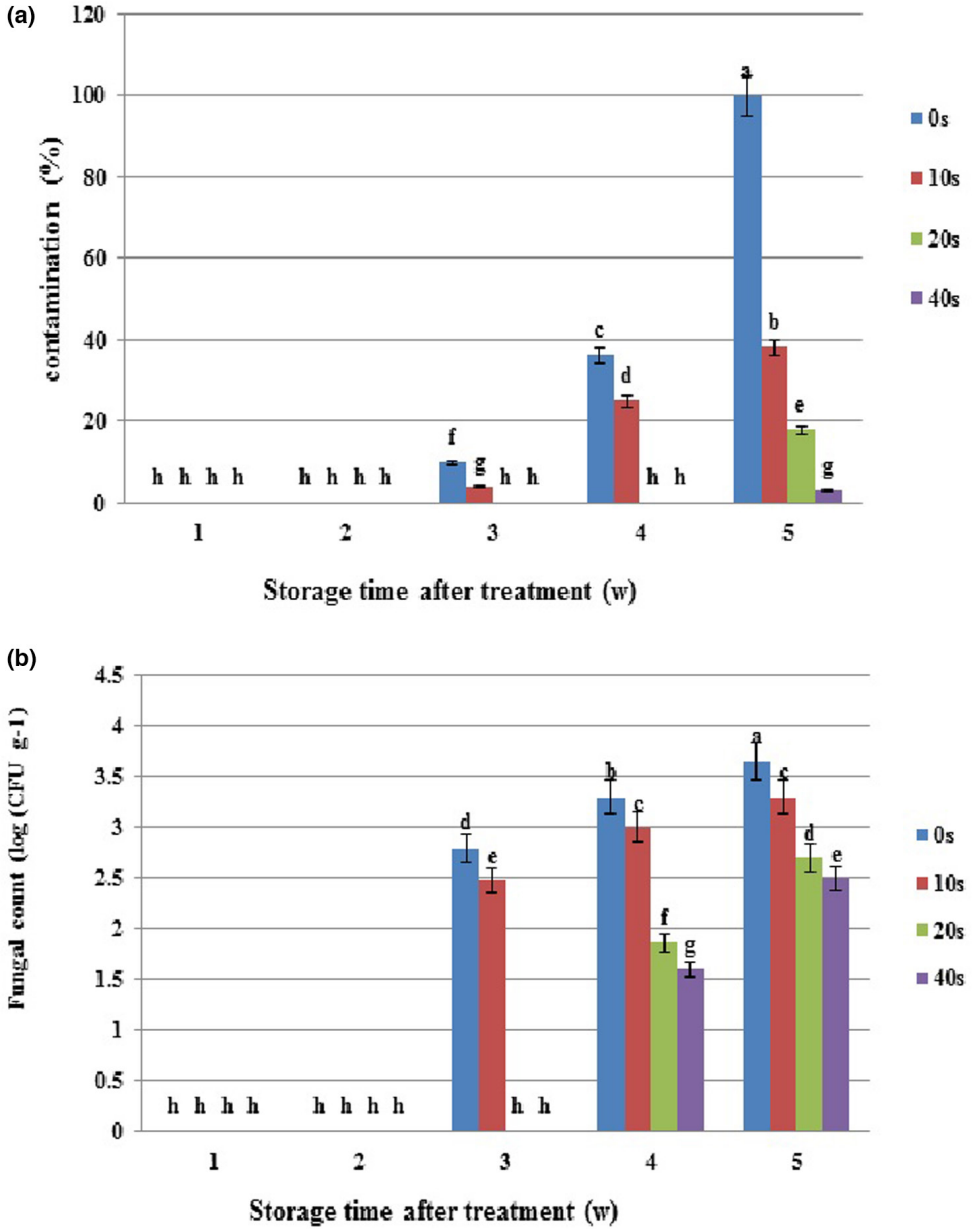
Microbial population and natural decay of grapes with cold plasma treatment (0, 10, 20, and 40 s) during storage at 4°C. (a) Contamination (%) and (b) Fungal count of treated grapes. Values are the mean of three replications. Column labeled with different small letters (a–h) indicates significant differences among different cold plasma treatments during storage time (*p* ≤ .05). Vertical bars depict standard error.

**FIGURE 3:**
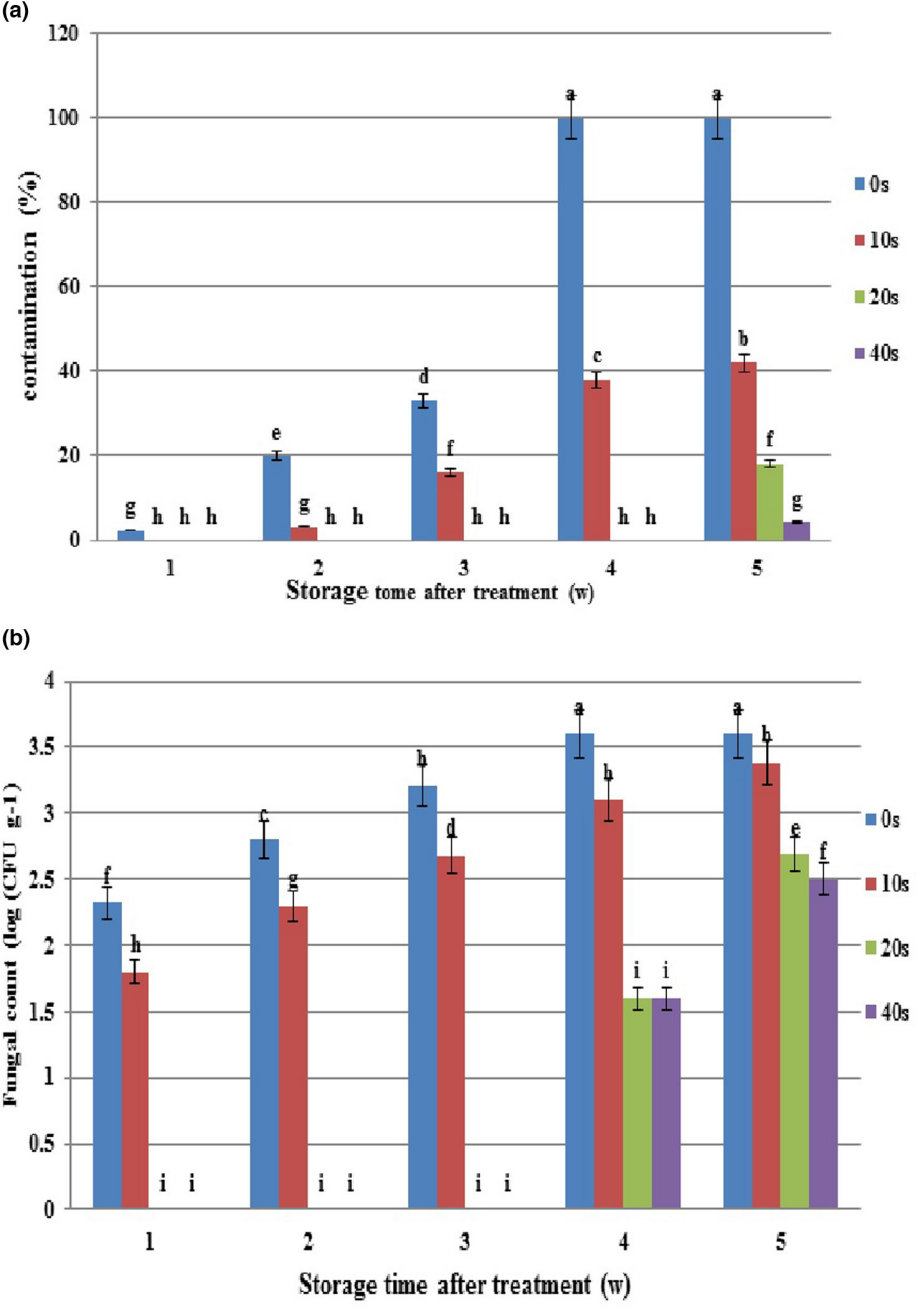
Microbial population and natural decay of grapes inoculated with Botrytis after cold plasma treatment (0, 10 20, and 40 s) during storage at 4°C. (a) Contamination (%) and (b) Fungal count of treated grapes. Values are the mean of three replications. Column labeled with different small letters (a–h) indicates significant differences among different cold plasma treatments during storage time (*p* ≤ .05). Vertical bars depict standard error.

Plasma application during storage significantly reduced the infection rate and microbial load in healthy and *Botrytis*‐inoculated samples (Figures 2, 3a,b).

**FIGURE 4:**
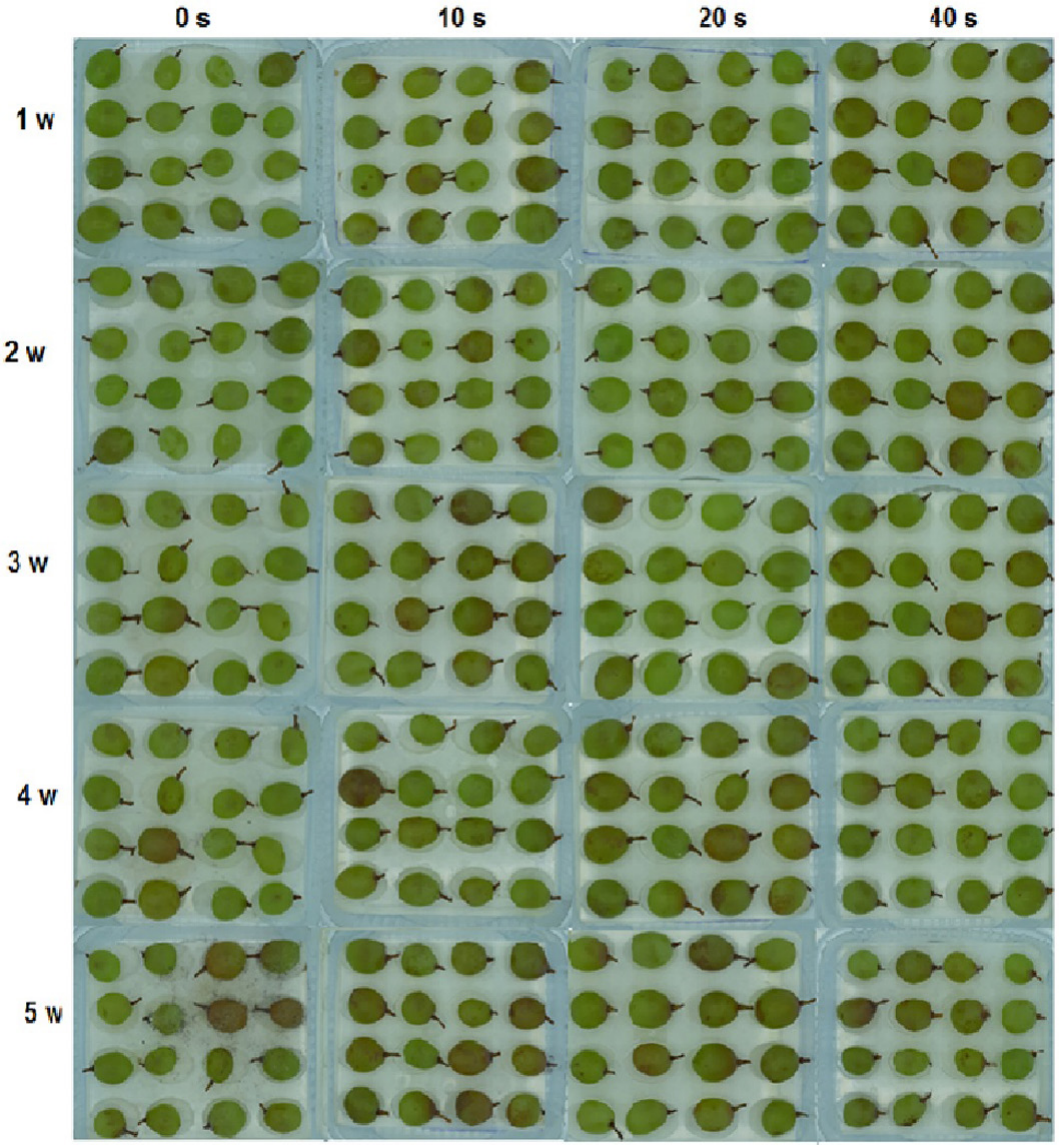
Physical images of grape with cold plasma treatment (0, 10 20, and 40 s) during storage (1, 2, 3, 4, and 5 weeks) at 4°C.

In the first and second experimental weeks, no signs of infection were observed in the control and samples in different plasma levels. In the third week, 10% and 4% infection rates were noticed in the control and 10‐s plasma treatment, respectively. However, the disease signs were absent in samples under 20‐ and 40‐s plasma treatments. Uptrend infection rates were recorded in the control (25%) and 10‐s plasma treatment (32%) in the fourth week while samples in 20‐ and 40‐s plasma treatments were still disease‐free in the same week. The disease was finally detected in 20‐ and 40‐s plasma treatments in the fifth week, when the control (100%) and 40‐s plasma (3.4%) presented the maximum and minimum infection rates, respectively. The same treatments showed the highest and lowest changes in infection spread, respectively (Figure 2a). Figure 4 depicts apparent changes in grapes during storage. Altogether, plasma application significantly reduced infection rates compared to the control, and increasing plasma application time from 10 to 40 s could more effectively reduce infection spread. As shown in Figure 2b, infection signs were not found in the control and plasma‐treated samples (CFU = 0) up to the second experimental week. In the third week, microbial load reached 2.7 and 2.5 log CFU g^−1^ in the control and 10‐s plasma, respectively, and 20‐ and 40‐s plasma samples were still free of the disease. Microbial load showed uptrends in the control and all plasma levels in the fourth and fifth weeks and reached its maximum value in the fifth week. Overall, the control and 40‐s plasma contained the highest (3.6 log CFU g^−1^) and lowest (2.5 log CFU g^−1^) microbial load values at the end of the experimental period. During storage, minimum changes in the microbial load belonged to 40‐sec plasma, indicating the plasma treatment efficiency in the control of the microbial load.

A similar trend was observed in the infection rate and microbial load of *Botrytis*‐inoculated samples compared to healthy fruits. However, the infection rate and microbial load were higher due to initial *Botrytis* inoculation, along with a more rapid spread of the disease.

**FIGURE 5:**
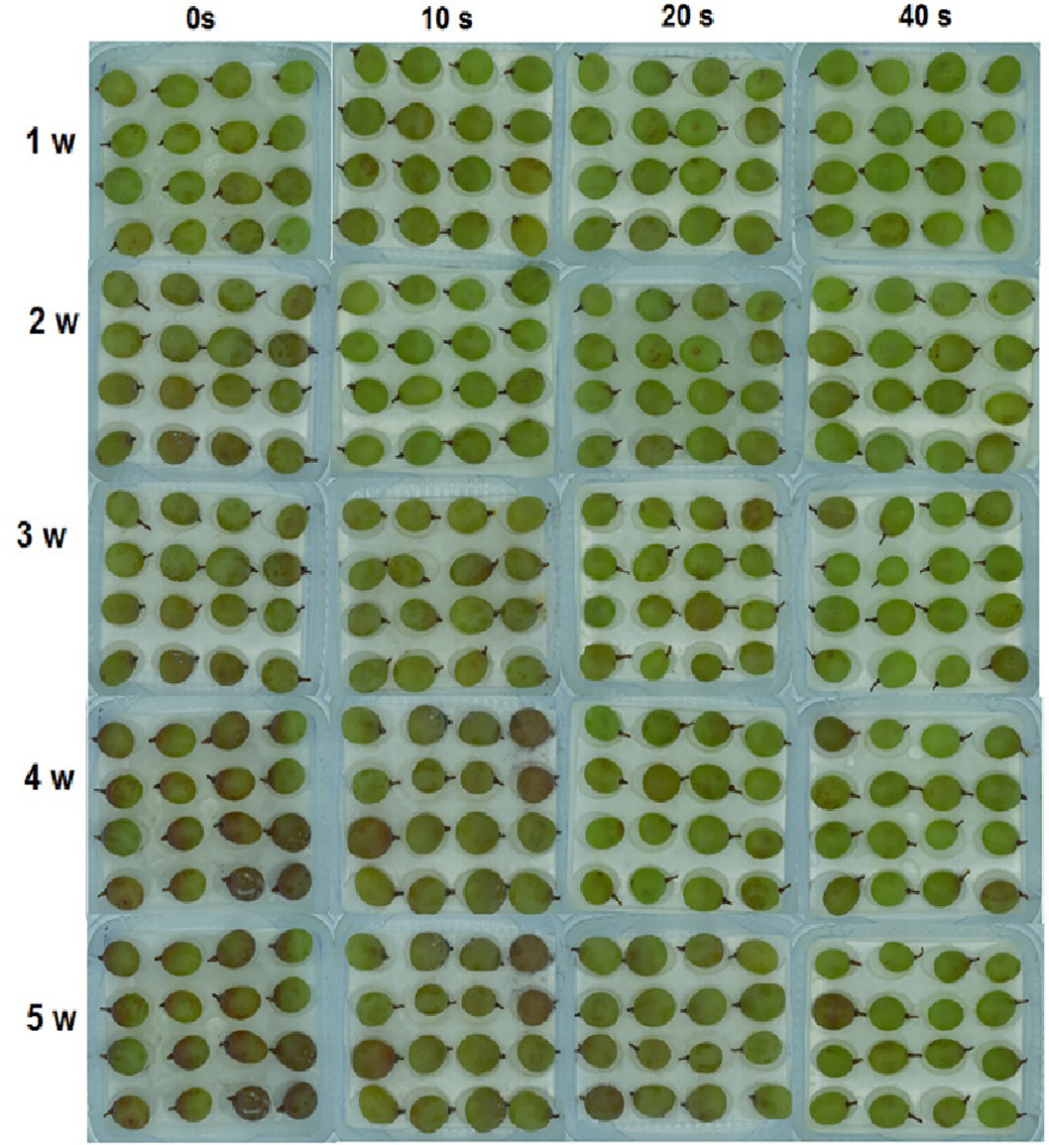
Physical images of grapes inoculated with Botrytis after cold plasma treatment (0, 10 20, and 40 s) during storage (1, 2, 3, 4, and 5 weeks) at 4°C.

Based on Figure 3a, the infection rate was 2% in the first experimental week whereas the disease signs were absent in samples treated with different plasma levels. In the second week, the infection rate increased in the control, and it was 5% in 10‐s plasma, but 20‐ and 40‐s samples were not still infected with the disease. The uptrend infection rate in the control (100%) and 10‐s plasma (41%) samples continued and reached the maximum level in the fifth week compared to baseline levels. Samples in 20‐ and 40‐s plasma treatments showed disease symptoms in the fifth week. The highest and lowest infection rates were recorded in the control (100%) and 40‐s plasma (4.5%) at the end of the experimental period. Figure 5 exemplifies grape apparent changes during storage.

Microbial load count increased in the control and different plasma levels in *Botrytis*‐inoculated samples during storage. In the first week, microbial loads were 2.4 log CFU g^−1^ and 1.7 log CFU g^−1^ in the control and 10‐s plasma. Over time, microbial loads increased in the control (3.6 log CFU g^−1^) and 10‐s plasma (3.3 log CFU g^−1^), with the highest level in the fifth week. Microbial loads (0 log CFU g^−1^) were recorded in 20‐ and 40‐s plasma treatments up to the third week, but they increased in the fourth week and reached maximum values in the fifth week (2.6 log CFU g^−1^ and 2.4 log CFU g^−1^, respectively). According to the results, plasma application reduced microbial load in comparison with the control. In all the examined times, the uppermost and lowermost microbial loads belonged to the control and 40‐s plasma, which contained the maximum (3.6 log CFU g^−1^) and minimum (2.5 log CFU g^−1^) microbial loads, respectively, at the end of the experimental period (Figure 3b).

Our results revealed that the disease symptoms emerged with more delay both in healthy and *Botrytis*‐inoculated samples in 20‐ and 40‐s plasma levels compared to the 10‐s level. In healthy samples, the disease in 10‐s plasma emerged in the third week concurrent with the control (10%), while *Botrytis*‐inoculated samples presented the disease in the second week (4%) only 1 week after the control. Although the disease concurrently occurred in 20‐ and 40‐s plasma levels, the infection rate was higher in the former than in the latter at the end of the experiment (Figures 2, 3a,b). Therefore, the use of 40‐s plasma treatment seems to be more effective in disease control. However, plasma application efficiency depends on maintaining crop quality in addition to the control of disease spread.

This observation corresponds to that of Bermúdez‐Aguirre et al. (2013) who studied the effect of plasma on lettuce and tomato plants and reported the highest microbial load reduction at the utmost plasma application time. In a study on the effect of dielectric plasma on strawberry samples, the total initial bacterial load and mold/yeast microbial load decreased after plasma treatment (Misra, Patil, et al., 2014). The APP ability in the inactivation of bacteria on the surfaces of apples, cantaloupes, and lettuce was reported by Critzer et al. (2007) and Segura‐Ponce et al. (2018). They also claimed a direct relationship between the reduction of microorganisms and increasing plasma application time at the end of the experiment. In the other study, cold plasma treatment led to the destruction of yeast, reduction of fungal microbial load, and gray rot caused by *Botrytis* in blueberries (Dong & Yang, 2019; Lacombe et al., 2015; Rana et al., 2020).

A variety of RONS (reactive oxygen and nitrogen species) are produced during the discharge of electrical charge during plasma generation (Hu et al., 2021). RONS are produced by creating oxidative stress in the cells and lead to oxidative damage in the cell membrane and intracellular components of the microorganism and finally their cell death (Dobrynin et al., 2009; Ma et al., 2016; Xu et al., 2020, 2021). On the other hand, cold plasma leads to the destruction of microorganisms by destroying DNA and guanine oxidation (Rana et al., 2020).

### 3.2 Crop quality assessment

#### 3.2.1 Physical properties

##### Weight loss

**FIGURE 6:**
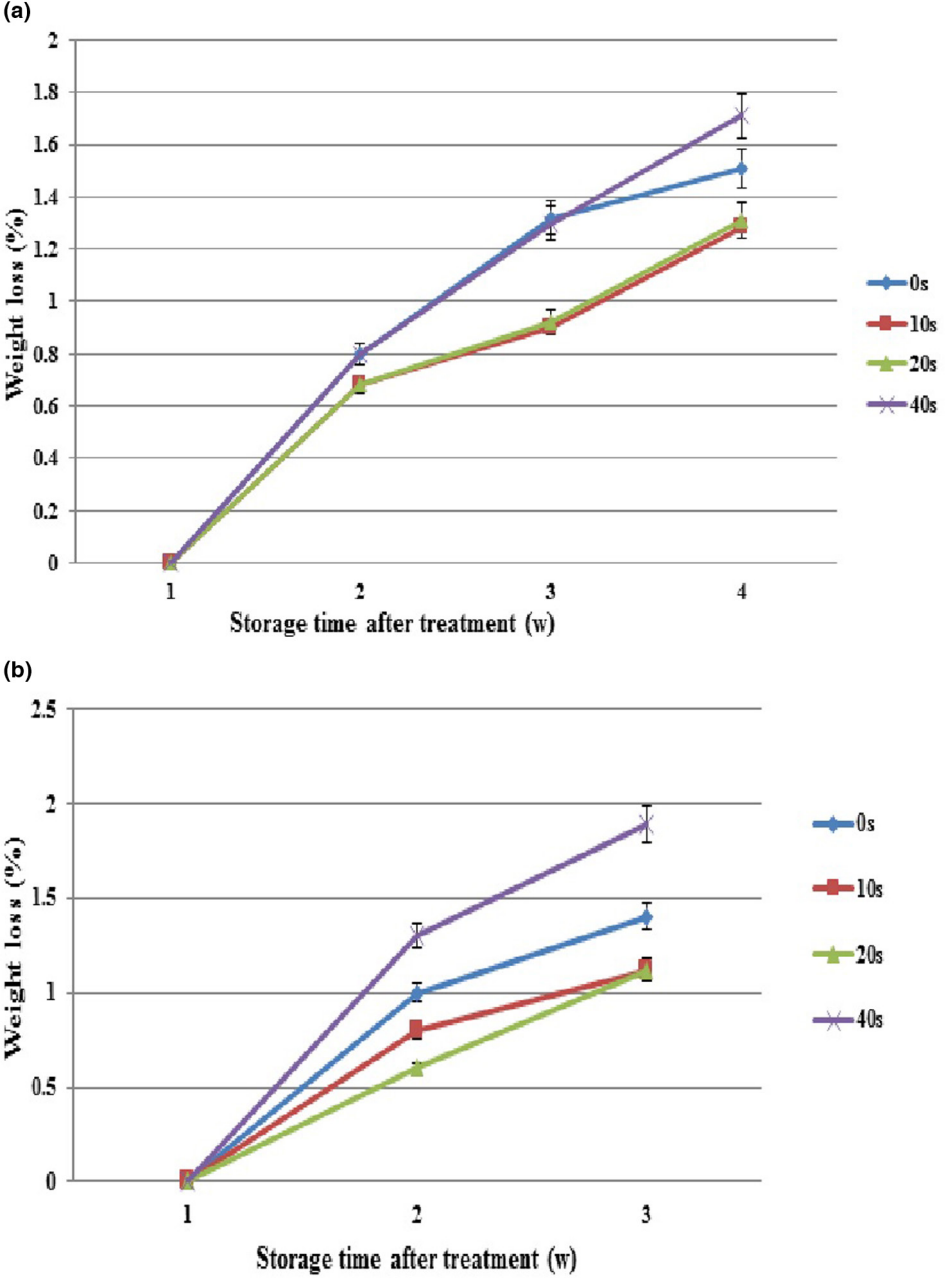
Weight loss of grapes with cold plasma treatment (0, 10 20, and 40 s) during storage at 4°C. (a) grapes without fungi inoculation and (b) grapes inoculated with Botrytis. Values are the mean of three replications. Vertical bars depict standard error.

Weight loss rates increased in healthy samples at different plasma levels during storage. Different plasma levels were not significantly different by the second experimental week. In the third and fourth weeks, weight loss rates increased significantly in the control and 40‐s plasma compared to 10‐ and 20‐s plasma. The highest weight loss (1.72) was observed in 40‐s plasma in the fourth (last) week when the lowest weight loss (1.7) occurred in 10‐s plasma, which was not significantly different from 20‐s plasma (Figure 6a).

With increasing time, weight loss rates increased in *Botrytis*‐inoculated samples in the control and all plasma levels. This rising trend in weight loss was higher in the control and 40‐s plasma treatment (1.9), showing the utmost level in the last experimental week. Although weight loss increased in all plasma levels over time, no significant difference was found between 10‐ and 20‐s plasma levels (Figure 6b).

Grapes contain high moisture due to their high internal water volume, making them very sensitive to storage conditions, during which crop moisture gradually exits the tissue, resulting in crop weight loss. Moreover, weight loss rates increase because of factors such as fungal infection and high temperatures. Since fruit cells have respiratory activity, some fruit water is allocated to this activity with increasing grape storage time (Lacombe et al., 2015).

During storage, weight loss increases in the control treatment because of the infection spread and crop aging. On the other hand, weight loss in 40‐s plasma treatment may result from fruit tissue destruction caused by plasma application time and moisture exit from the fruit peduncle. The lower weight loss rates in 10‐ and 20‐s treatments may be caused by the lower infection rate than the control and the lower destructive effect of plasma than 40‐s plasma.

A comparison between healthy and *Botrytis*‐inoculated samples revealed more weight loss in the latter group during the measurement because of the increased infection caused by initial fungal inoculation. The 20‐s treatment seems to positively affect weight loss control in addition to disease control.

Min et al. (2018) showed that the application of appropriate levels of cold plasma does not have a significant effect on the weight loss of tomatoes stored at 10 and 25°C. Cold plasma treatment significantly reduced microbial load in lettuce without affecting product weight loss (Song et al., 2015). In strawberries, plasma did not affect the percentage of fruit moisture after storage (Rana et al., 2020). These results were consistent with the findings of the present study.

##### Color changes

At all plasma levels, the lightness (L*) and b* indices decreased while the a* index increased in healthy samples over time (Table 1). The lowest L* (32.4) and b* (10.9) indices and the highest a* index (3.3) were recorded in 40‐s plasma treatment at the end of the experimental period. Decreased L* and b* indices and an elevated a* index were observed in *Botrytis*‐inoculated samples during storage. A comparison between plasma levels and the control showed no significant differences in colorimetric indices during 3 weeks, but 40‐s plasma was significantly different from 10‐ and 20‐s levels in colorimetric indices in the fourth week (Table 1).

**TABLE 1:** Color parameter (L*, a*, and b* values) of grapes with cold plasma treatment (0, 10, 20, and 40 s) during storage at 4°C.

	Storage time (weeks after treatment)							
Color parameter	Treatment time (s)	Without fungi inoculation				Botrytis inoculation		
		1	2	3	4	1	2	3
L*	0	39.3^a^	38.5^ab^	37.9^bc^	36.9^de^	35.1^a–c^	33.2^b–d^	31.6^d^
	10	39.1^a^	38.4^ab^	37.8^b–d^	35.9^fg^	35.5^ab^	32.6^cd^	31.7^d^
	20	38.4^ab^	36.8^ef^	35.5^g^	33.6^h^	36.1^a^	33.6^a–d^	32.0^d^
	40	36.4^e–g^	37.1^c–e^	34.3^h^	32.4^i^	34.9^a–c^	33.2^b–d^	31.7^d^
a*	0	−1.2^ab^	−1.9^a–c^	−2.5^bc^	−2.6^bc^	−2.5^ab^	−3.3^c^	−3.4^c^
	10	−1.3^ab^	−1.8^a–c^	−2.2^a–c^	−2.7^bc^	−2.4^ab^	−2.9^cb^	−3.6^c^
	20	−1.4^ab^	−1.6^ab^	−0.78^a^	−2.7^bc^	−2.4^ab^	−3.2^bc^	−3.5^c^
	40	−1.5^ab^	−2.1^a–c^	−2.49^a–c^	−3.396^c^	−1.7^a^	−2.5^ab^	−3.6 ^c^
b*	0	13.5^c^	13.2^dc^	12.59^c–e^	10.95^f^	16.1^a^	14.3^a–c^	13.6^a–c^
	10	13.3^c^	12.6^c–e^	11.54^ef^	11.36^er^	15.3^ab^	15.0^a–c^	12.7^bc^
	20	13.8^c^	12.7^c–e^	11.61^ef^	11.02^f^	15.7^aa^	14.7^a–c^	13.3^a–c^
	40	17.1^a^	15.5^b^	11.94^d–f^	10.95^f^	15.5^ab^	14.6^a–c^	12.2^c^

*Note*: Data presented as mean ± SD. Values followed by different small letters (a–f) in the same column (1–4 for without fungi inoculation and 1–3 for Botrytis inoculation) are significantly different (*p* ≤ .05).

**FIGURE 7:**
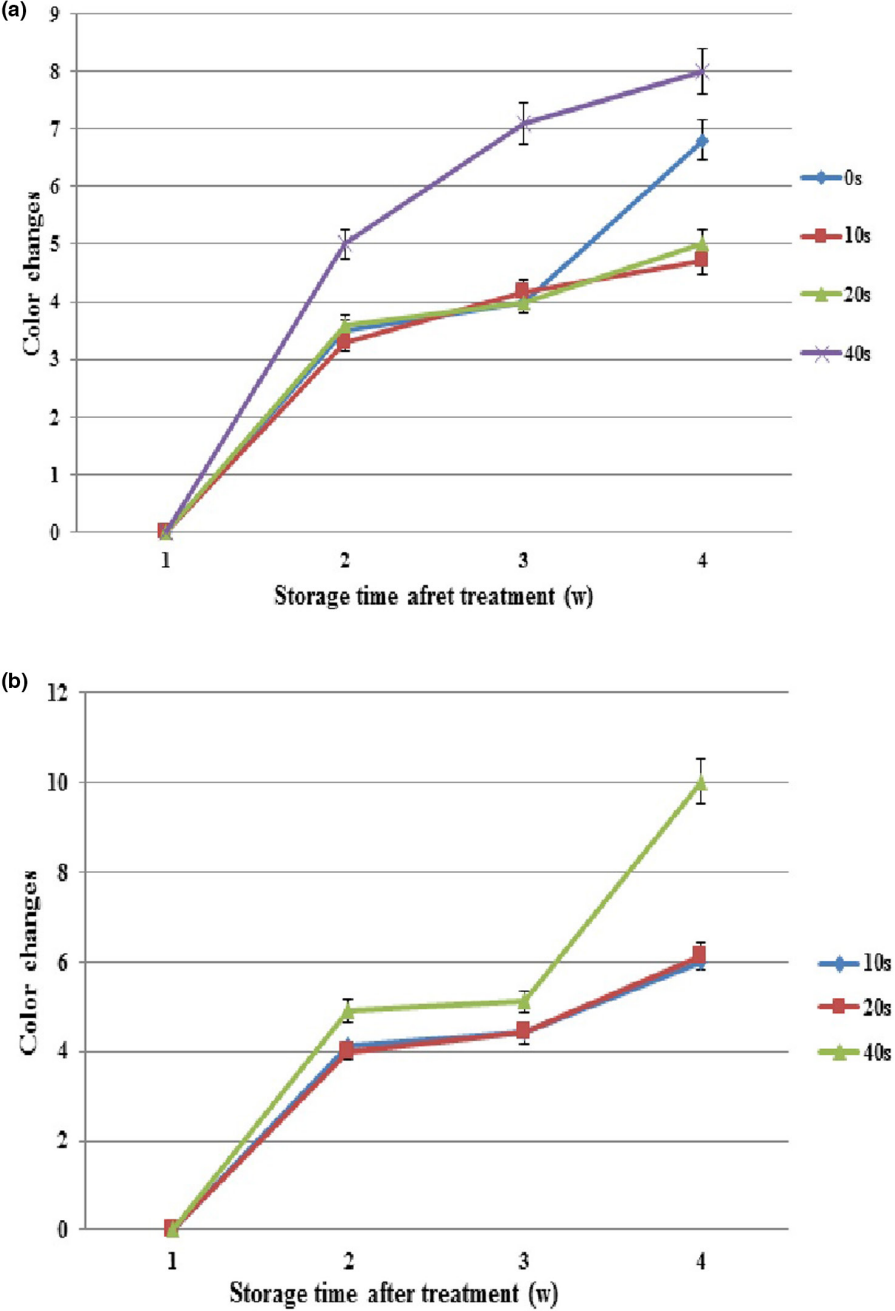
Color changes of grapes with cold plasma treatment (0, 10 20, and 40 s) during storage at 4°C. (a) grapes without fungi inoculation and (b) grapes inoculated with Botrytis. Values are the mean of three replications. Vertical bars depict standard error.

Reductions in L* and b* indices and an increase in a* index cause blackness, increase greenness, and reduce yellowness in the crop. In this research, changes in colorimetric indices led to color changes in the treated samples during storage. Based on Figure 7a, color changes increased significantly in healthy samples at all plasma levels over time. The plasma levels of 0, 10, and 20 s were not significantly different during the first 3 weeks. In the fourth week, color changes in the control were significantly different from those of 10‐ and 20‐s treatments. The highest color changes were observed in 40‐s plasma (8) at the end of the experiment compared to the control (6.8), whereas the changes were considerably lower in 10‐ and 20‐s plasma treatments.

In *Botrytis*‐inoculated samples, the interaction of storage period (3 weeks) in 10‐, 20‐, and 40‐s levels significantly affected the color change index, but 0‐, 10‐, 20‐, and 40‐s plasma levels were not significantly different during 3 weeks. Color changes increased in all plasma levels during storage, and the highest color changes belonged to 40‐sec plasma (9.9) at the end of the experiment. Color changes were not significantly different between 10‐ and 20‐s plasma treatments (Figure 7b).

Since plasma treatment is only applied to the crop surface where most reactions occur, the appearance and physical assessment can be very effective. Any change in the crop color and tissue can indicate a chemical reaction in the crop. The radiations from plasma have a short life and propagation power, so their effect is generally superficial and affects the appearance of the product such as color (Xu et al., 2021). Also, the intensity and duration of plasma application can affect the color changes of the product. In this study, no significant changes occurred in the control and 10‐ and 20‐s plasma levels before the spread of fungal infections. Similarly, Bermúdez‐Aguirre et al. (2013) reported no significant changes in color indices and color changes in tomato, lettuce, and carrot crops. Misra, Keener, et al. (2014) also observed no significant effect of plasma application on the color changes of strawberry samples compared to the control.

As mentioned above, plasma application time affects maintaining the crop color. In the current investigation, the L*, a*, and b* indices (thus the crop color) changed significantly with increasing plasma application time to 40 s. Akbarian et al. (2019) studied the effect of NTP on saffron quality properties and reported that changes in plasma application time and the voltage level significantly reduced redness, increased yellowness, and changed saffron powder color. Color changes caused by plasma application can result from pigment oxidation and degradation by reactive species with increasing voltage (Bermúdez‐Aguirre et al., 2013). The researchers attributed color changes resulting from plasma application to ozone and hydroxyl radicals produced by NTP as their levels rise with increasing time, which increases pigment oxidation and degradation, thereby lowering pigments (Jayasena et al., 2015; Ramazzina et al., 2015; Vukić et al., 2017). NTP treatment for more than 60 s causes color changes in blueberries (Lacombe et al., 2015). In blueberry, the inappropriate duration of plasma treatment causes the loss of the surface wax of the fruit and leads to the darkening of the product (Hu et al., 2021). Therefore, optimal plasma application time with a suitable voltage can effectively improve the efficiency of this technique.

#### 3.2.2 Mechanical properties

**FIGURE 8:**
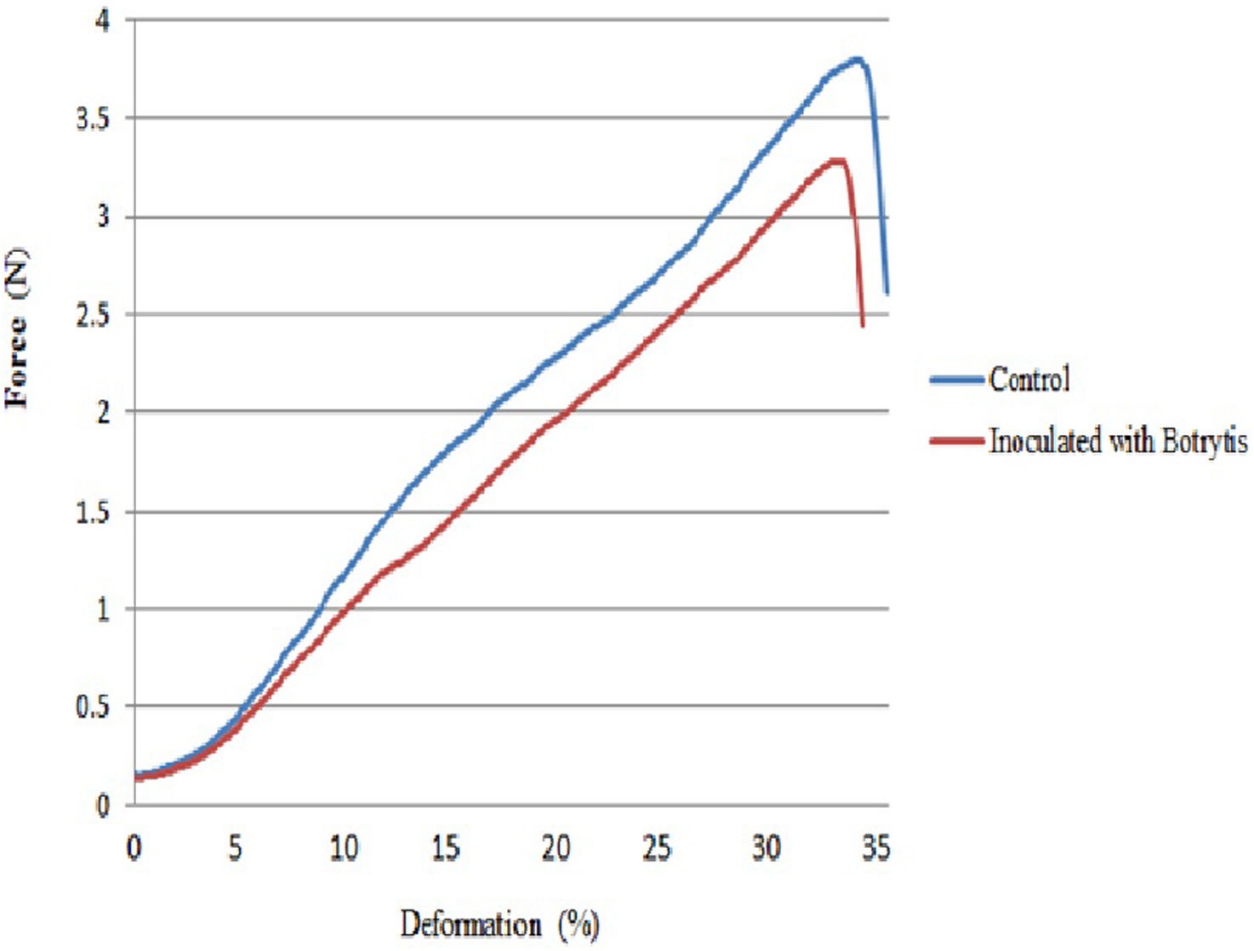
Force‐deformation curve of puncture test for control grape sample (without fungi inoculation) and grapes inoculated with Botrytis after cold plasma treatment.

Figure 8 shows the displacement–power curve drawn for the control and *Botrytis*‐inoculated samples after plasma application. In healthy samples, different plasma levels were not significantly different in penetration power and the required energy. In 40‐s plasma treatment, the modulus of elasticity value (0.0088 GPa) was significantly lower than the other plasma levels (Table 2). A comparison of the simple effect of the storage period indicated that mechanical indices decreased over time, and the lowest values of modulus of elasticity (0.0063 GPa), penetration power (2.45 N), and required energy (8.05) were measured in the last week (Table 2).

**TABLE 2:** Mechanical parameter (Emod, Fmax, and *W*) of grapes with cold plasma treatment (0, 10, 20, and 40 s) during storage at 4°C.

	Mechanical parameter		
	Emod	Fmax	*W*
Treatment time (s)			
0	0.0105^a^	3.84^ab^	13.59^ab^
10	0.0105^a^	3.94^ab^	13.57^ab^
20	0.0107^a^	4.21^a^	15.02^a^
40	0.0088^b^	3.49^b^	11.67^b^
Storage time (weeks after treatment)			
1	0.0141^a^	5.31^a^	21.78^a^
2	0.0118^b^	4.3^b^	15.1^b^
3	0.0083^c^	3.44^c^	9.96^c^
4	0.0063^d^	2.45^d^	8.02^d^

*Note*: Data presented as mean ± SD. Values followed by different small letters (a–d) in the same column are significantly different (*p* ≤ .05).

The modulus of elasticity, penetration power, and required energy showed downtrends in *Botrytis*‐inoculated samples at all plasma levels during storage. As shown in Table 3, the control and 10‐ and 20‐s plasma treatments were not significantly different by the second week of storage. At the end of the experiment, these parameters decreased in the control due to the spread of tissue stiffness infection compared to 10‐ and 20‐s plasma treatments. The maximum changes in tissue stiffness reduction occurred in 40‐s plasma treatment in which penetration power (1.78 N) and required energy (3.18) reached the minimum values (Table 3).

**TABLE 3:** Mechanical parameter (Emod, Fmax, and *W*) of grapes inoculated with Botrytis after cold plasma treatment (0, 10, 20, and 40 s) during storage at 4°C.

Mechanical parameter	Treatment time (s)	Storage time (weeks after treatment)		
		1	2	3
Emod	0	0.0130^a^	0.0092^b^	0.0059^d^
	10	0.0133^a^	0.0092^b^	0.0076^c^
	20	0.0133^a^	0.0092^b^	0.0077^c^
	40	0.0092^b^	0.0068^c^	0.0028^e^
Fmax	0	5.53^a^	4.57^b^	2.09^d^
	10	5.62^a^	4.4^b^	3.60^c^
	20	5.79^a^	4.63^b^	3.57^c^
	40	3.3^c^	1.92^d^	1.78^e^
*W*	0	25.98^a^	17.09^b^	10.21^c^
	10	26.23^a^	17.09^b^	9.61^c^
	20	26.69^a^	16.45^b^	10.06^c^
	40	9.05^c^	4.52^d^	3.18^d^

*Note*: Data presented as mean ± SD. Values followed by different small letters (a–d) during storage (1–3 weeks) are significantly different (*p* ≤ .05).

The tissue is a major quality parameter in evaluating shelf life and consumer acceptance. Fruit tissue softness occurs because of cell wall destruction by lysing enzyme activity. Moreover, fungal infection causes tissue aging and cell wall breakage, thereby lowering tissue stiffness and indicating crop aging over time. The loss of crop weight and moisture can also accelerate the aging process and reduce the quality of mechanical properties (Rux et al., 2015). Reductions in the modulus of elasticity and penetration power indicate reduced crop quality and mechanical properties during storage, ultimately leading to crop aging.

In this study, tissue stiffness was not significantly different between the control and 10‐ and 20‐s plasma levels up to the middle of the storage period, but tissue stiffness decreased in the control with the infection spread. Likewise, Ahmadnia et al. (2021) presented evidence that plasma application led to no destructive effect on tissue stiffness in strawberries. An evaluation of the total results demonstrated stiffer tissues in plasma‐treated samples than in the control group. This observation may result from the reduced surface microorganism in plasma‐treated samples that increase the storage period of strawberry fruits. In a study on the effect of dielectric barrier discharge (DBD) plasma on the physicochemical parameters of sliced kiwi fruits, no significant differences were found between the control and plasma‐treated groups (Ramazzina et al., 2015). Tappi et al. (2016) exposed melon slices to DBD plasma and measured tissue stiffness. They reported no significant differences between the control and plasma‐treated groups.

In our study, increasing plasma application time to 40 s reduced grape stiffness more than those of the control and the other plasma levels. Ahmadnia et al. (2021) found no destructive effect of plasma application on tissue stiffness. However, changes in the plasma application work cycle can reduce tissue stiffness in strawberries. Misra, Patil, et al. (2014) reported that plasma application could reduce tissue stiffness. In blueberry, cold plasma treatment for less than 15 min had no effect on the firmness of the tissue, but 20 min of plasma led to the softness of the fruit (Hu et al., 2021). Therefore, it is of paramount importance to optimize plasma application time and intensity. The negative effect of inappropriate plasma levels on the reduced strawberry stiffness was attributed to the produced ozone levels (Aday et al., 2013).

The reduced crop stiffness after 40‐s plasma treatment might also result from weight loss. Softness and reduced stiffness in fresh crops are directly related to reduced water content and crop weight (Rux et al., 2015). Tissue softness in the control and 40‐s plasma treatments can be attributed to decreased moisture and weight loss. Crop stiffness in the control sample because of the infection spread in 40‐s plasma treatment at the end of the experiment due to tissue degradation and moisture exit from the peduncle‐fruit connection site.

#### 3.2.3 Crop temperature

**FIGURE 9:**
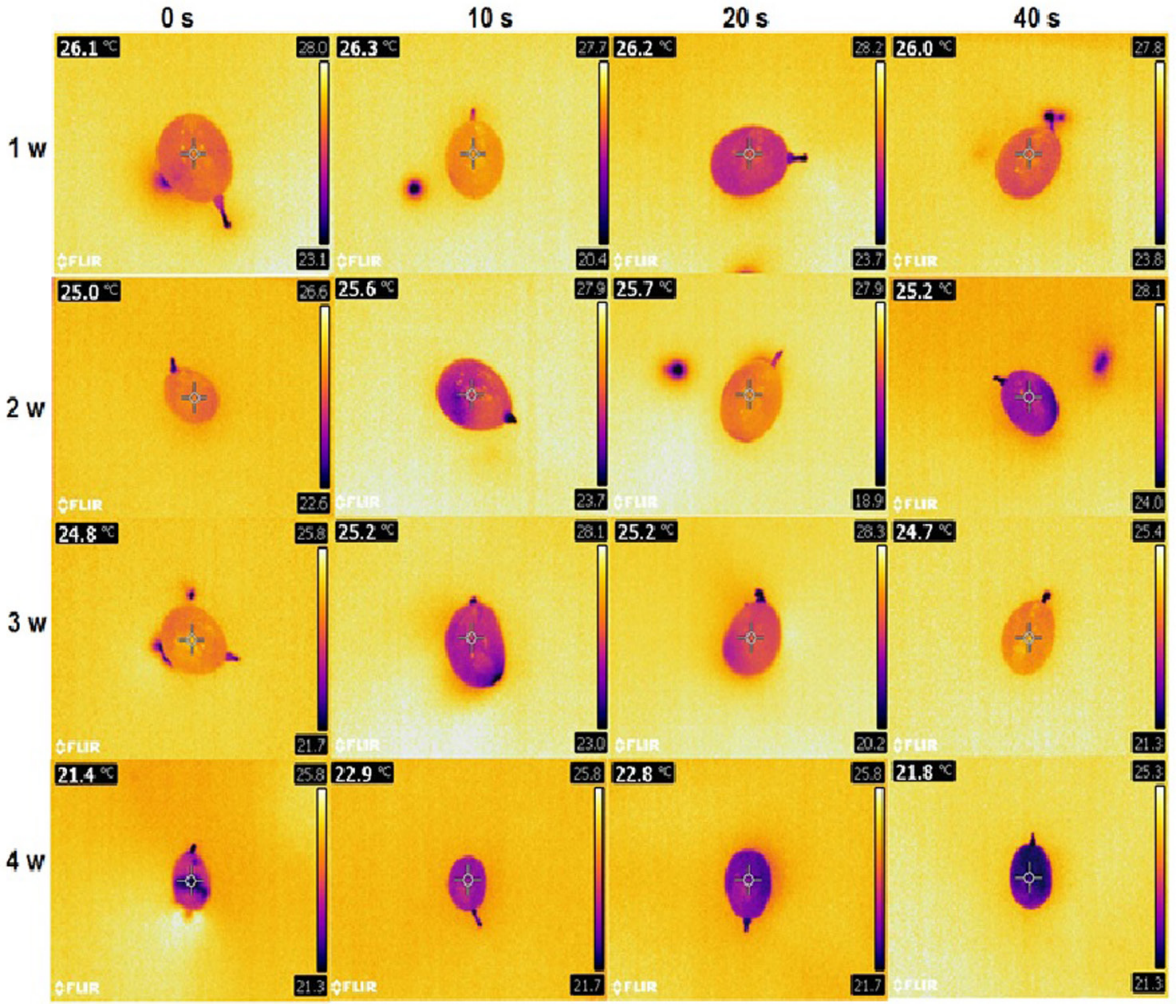
Thermal images of grapes after cold plasma treatment (0, 10, 20, and 40 s) during storage (1–4 weeks) at 4°C.

Downward crop temperatures were detected in healthy samples at all plasma levels during storage. At all studied times, crop temperatures were lower in the control and 40‐s plasma groups than 10‐ and 20‐s levels. There were no significant differences between the control and 40‐s plasma groups. The same condition was also noticed between 10‐ and 20‐s levels at all studied times. The least temperatures were recorded in the control (21.83°C) and 40‐s (21.4°C) plasma groups compared to mean temperatures of 22.9 and 22.9°C in 10‐ and 20‐s treatments, respectively. The highest temperature changes were observed in 40‐s plasma (4.13°C) and control (4.9°C) groups. Temperature changes were 4.23°C and 4.1°C in 10‐ and 20‐s plasma treatments, which generally showed lower temperature reductions than the control and 40‐s plasma groups (Figure 9).

**FIGURE 10:**
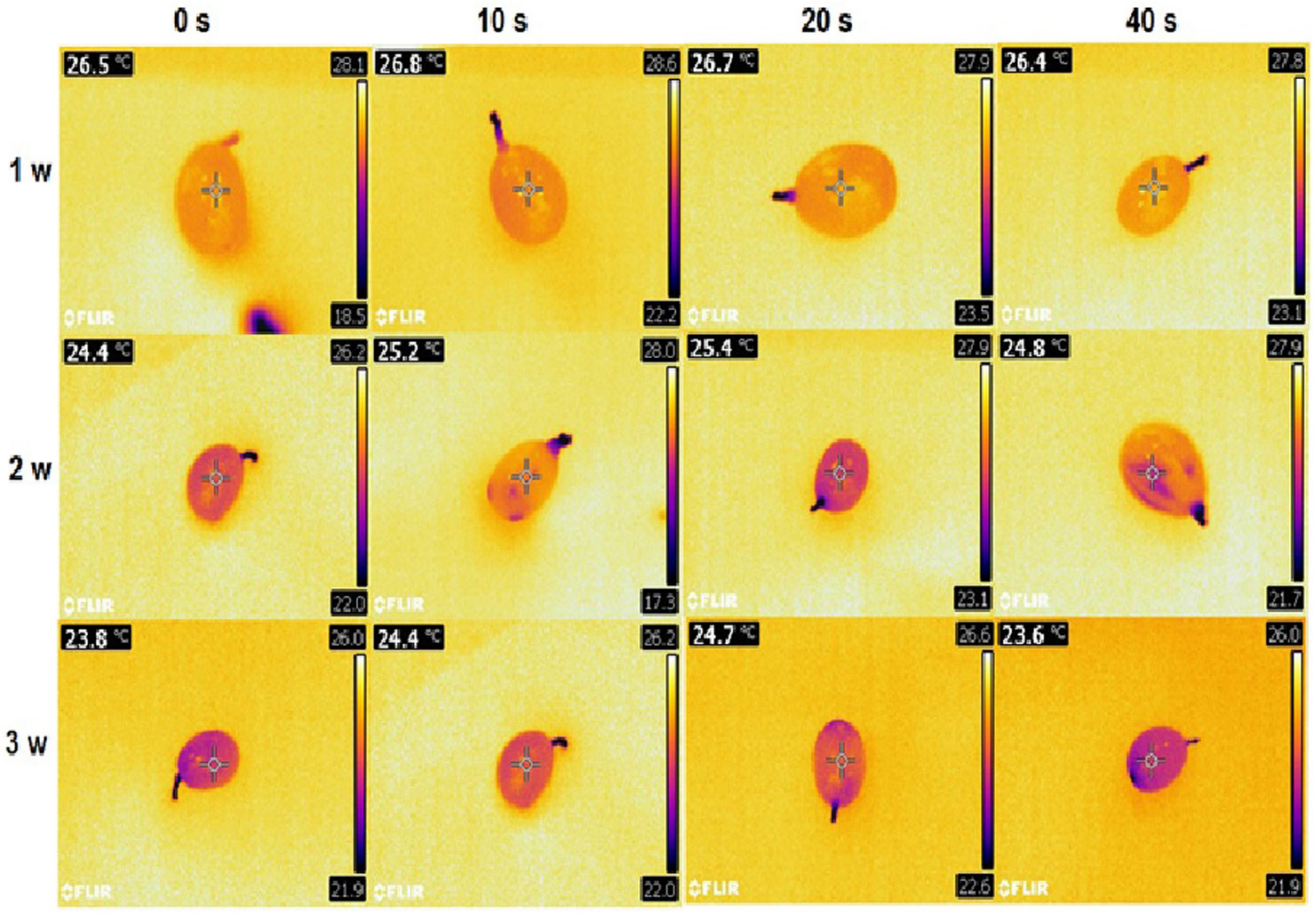
Thermal images of grapes inoculated with Botrytis after cold plasma treatment (0, 10, 20, and 40 s) during storage (1–4 weeks) at 4°C.

In *Botrytis*‐inoculated samples, crop temperatures decreased at all plasma levels with increasing time. The most temperature changes from 26.46 to 23.8°C occurred in the control sample during 3 weeks (a reduction of 2.96°C). The lowest temperature decrease (from 26.83 to 24.4°C, with 2.13°C reduction) was measured in 10‐sec plasma treatment. The different plasma levels were not significantly different in the first week, but a different pattern was observed in the second and third weeks when mean temperatures in the control and 40‐s plasma groups showed no statistically significant difference (Figure 10).

Weight loss and moisture reduction reduce the crop surface temperature, a process that mostly occurs in treatments with greater infection spread. This results from an increase in the infection resulting in moisture reduction and elevated weight loss, followed by a reduction in the crop temperature. In this study, the minimum temperature reduction occurred in 10‐ and 20‐s plasma treatments. Higher temperature reductions were recorded in the control because of infection spread and weight loss and in 40‐s plasma samples due to weight loss resulting from the destructive effect of plasma.

To evaluate crop temperature, thermal imaging is a good technique for quality assessment and the examination of crop microbial contamination. According to Hu et al. (2021), cold plasma can increase blueberry skin temperature up to only 34.6°C, so this method is considered as a nonthermal theology. Our results showed that a further reduction in the crop temperature occurs with more weight loss and infection rates in the samples. In other words, temperature decreases with higher tissue degradation rates. Any factor that causes crop aging and infection spread will reduce its temperature as well (Varith et al., 2003; Hellebrand et al., 2002; Chelladurai et al., 2010).

#### 3.2.4 Chemical properties

TSS and pH rose and TA declined in both healthy and *Botrytis*‐inoculated samples at all plasma levels over time (Table 4). In healthy samples, pH values were lowermost in the first experimental week and different plasma levels were not significantly different in this index. At all plasma levels, pH values increased with time and were maximized in the last week. This rising trend was lower in 40‐s plasma treatment over time, with a minimum pH (3.98) in the last week while the maximum pH (4.11) was measured in the control (Table 4).

**TABLE 4:** Chemical parameter (TSS, pH, and TA) of grapes after cold plasma treatment (0, 10, 20, and 40 s) during storage at 4°C.

Chemical parameter	Storage time (weeks after treatment)							
	Treatment time (s)	Without fungi inoculation				Botrytis inoculation		
		1	2	4	1	2	3
TSS	0	22.33^efg^		22.75^ef^	24.71^b^	19.5^f^	21. 67^d^	24.04^ab^
	10	22.13^fg^		22.91^de^	23.90^c^	18.8^f^	21.16^d^	23.11^c^
	20	22.03^g^		22.80^def^	24.00^c^	19.69^ef^	20.90^d^	23.06^c^
	40	22.32^efg^		22.80^def^	25.63^a^	19.59^ef^	20.47^de^	24.28^a^
pH	0	3.52^e^		3.86^sd^	4.11^a^	3.51^c^	3.70^b^	3.87^a^
	10	3.54^e^		3.89^bcd^	4.00^abc^	3.41^c^	3.67^b^	3.81^a^
	20	3.51^e^		3.86^cd^	4.00^abc^	3.50^c^	3.78^b^	3.90^a^
	40	3.54^e^		3.85^d^	3.98^abcd^	3.52^c^	3.75^b^	3.89^a^
TA	0	1.17^a^		0.83^ef^	0.64^h^	1.20^ab^	1.05^bc^	0.80^e^
	10	1.06^abc^	0.97^bcd^	0.76^fgh^	0.65^h^	1.06^bc^	0.98^cd^	0.83^de^
	20	1.08^ab^	0.95^cd^	0.78^fg^	0.65^h^	1.18^ab^	0.95^cde^	0.85^de^
	40	1.13^a^	0.88^def^	0.79^fg^	0.72^g^	1.24^a^	1.05^bc^	0.94^cde^

*Note*: Data presented as mean ± SD. Values followed by different small letters (a–h) in the same column (1–4 for without fungi inoculation and 1–3 for Botrytis inoculation) are significantly different (*p* ≤ .05).

TSS increased continuously at all plasma levels during storage (Table 4). This rising trend was significantly higher in the control and 40‐s plasma groups than in 10‐ and 20‐s plasma treatments. TSS was minimal (22.03) in 10‐s plasma treatment during the first experimental week, but there were no statistical differences between different plasma levels at this time. As reported above, TSS increased at all plasma levels over time, but this uptrend was considerably greater in the control (24.71) and 40‐s (25.63) plasma groups than in 10‐ and 20‐s plasma treatments at the end of the experiment. At this time, TSS values were not significantly different in 10‐ (24) and 20‐s (23.9) plasma treatments (Table 4).

TA levels declined gradually with time at all plasma levels and reached the minimum level in the last experimental week when the least TA level (0.64) belonged to the control, but there were no statistically significant differences with 10‐ and 20‐s plasma treatments. The TA level was maximal in the 40‐s plasma group (0.72) in the last experimental week (Table 4).

In *Botrytis*‐inoculated samples, the highest pH value (3.89) was recorded in the last (3rd) experimental week. A comparison of the interaction of plasma levels and storage period showed significant changes in TA and TSS with increasing time (Table 4), i.e., a reduction and an elevation occurred in the former and the latter, respectively. The control treatment contained the least TA level (0.8) in the last experimental week. At this time, different plasma treatments were not different significantly. The TSS level showed an upward trend at all plasma levels, and the greatest rise of TSS occurred in the control (24.04) and 40‐s (24.28) plasma groups in the last week. At this time, 10‐ and 20‐s plasma treatments contained the lowest TSS levels (23.6 and 23.11, respectively) with no statistically significant difference (Table 4).

An increase in pH during storage changes crop nature from an acidic to an alkaline state, which accelerates crop decay. A rise in respiration during storage changes pH to alkaline and increases crop decay. Such changes may result from an increase in respiration, infection spread, and an accelerated aging process, caused by the use of organic acids.

The elevated TSS levels during storage may be due to a high juice concentration because of crop water loss and weight reduction (Kader & Watkins, 2000). The increased TSS and pH levels during storage result in reduced levels of TA. The utmost pH value was observed in the control at all studied times. This result can be attributed to the infection spread in the control because fungal infections cause alkalinity in the environment. This trend was noticed in all healthy and *Botrytis*‐inoculated samples. The minimum pH value in 40‐s plasma treatment may result from the high effect of plasma on infection control.

In all healthy and *Botrytis*‐inoculated samples, maximum TSS levels were observed in the control and 40‐s plasma treatments, which may be caused by weight loss resulting from moisture reduction in 0‐ and 40‐s plasma treatments. This moisture reduction apparently increases the TSS index.

Previous research has shown that the effect of plasma is generally on the surface characteristics of the product, and in the appropriate voltage and duration, it has no effect on the internal chemical properties of the product (Hu et al., 2021). Our results correspond to those obtained in other crops. In blueberries, less than 15 min of plasma after 10 days, and in strawberries, 15 min of cold plasma with packaging after 5 days of storage had no effect on pH and TSS (Hu et al., 2021; Rana et al., 2020). Plasma application did not affect the chemical properties of water‐immersed button mushrooms (*Agaricus bisporus* L.), nor the pH of tomatoes (Misra, Patil, et al., 2014; Xu et al., 2016). The chemical properties of sliced kiwi fruits did not significantly change with NTP treatment (Ramazzina et al., 2015). NTP application in orange, tomato, and apple juices did not significantly affect pH values (Dasan & Boyaci, 2018).

## 4 CONCLUSION

The present research showed for the first time that NTP is an effective grape postharvest technology in reducing the decay caused by *Botrytis cinerea* during storage. In addition, short‐time NTP (≤20) could preserve grape quality, while long‐time NTP (40 s) could cause damages leading to grape losing weight, changing color, and softening. Additional investigations in future to measure the chemical compounds and secondary metabolites of grapes treated with NTP will lead to more efficiency in the use of plasma as a postharvest technology in grape.

## AUTHOR CONTRIBUTIONS

**Ali Khalaj:** Data curation (equal); methodology (equal); software (equal). **Ebrahim Ahmadi:** Methodology (equal); project administration (equal); supervision (equal); writing – review and editing (equal). **Sohiela Mirzaei:** Data curation (equal); methodology (equal); resources (equal). **Fahiemeh Ghaemizadeh:** Investigation (equal); validation (equal); writing – original draft (equal).

## FUNDING INFORMATION

This study was supported by the office of vice chancellor for research at Bu‐Ali Sina University (Thesis No. 6375).

## CONFLICT OF INTEREST STATEMENT

The authors declare that they have no known competing financial interests or personal relationships that could have appeared to influence the work reported in this paper or any conflict of interest.

## ETHICS STATEMENT

This study does not involve any human or animal testing.

## Data Availability

The data that support the findings of this study are available from the corresponding author upon reasonable request.
